# Transmembrane Domain 3 (TM3) Governs Orai1 and Orai3 Pore Opening in an Isoform-Specific Manner

**DOI:** 10.3389/fcell.2021.635705

**Published:** 2021-02-11

**Authors:** Adéla Tiffner, Lena Maltan, Marc Fahrner, Matthias Sallinger, Sarah Weiß, Herwig Grabmayr, Carmen Höglinger, Isabella Derler

**Affiliations:** JKU Life Science Center, Institute of Biophysics, Johannes Kepler University Linz, Linz, Austria

**Keywords:** Orai1, Orai3, STIM1, isoform-specific activation, CRAC channel

## Abstract

STIM1-mediated activation of calcium selective Orai channels is fundamental for life. The three Orai channel isoforms, Orai1-3, together with their multiple ways of interplay, ensure their highly versatile role in a variety of cellular functions and tissues in both, health and disease. While all three isoforms are activated in a store-operated manner by STIM1, they differ in diverse biophysical and structural properties. In the present study, we provide profound evidence that non-conserved residues in TM3 control together with the cytosolic loop2 region the maintenance of the closed state and the configuration of an opening-permissive channel conformation of Orai1 and Orai3 in an isoform-specific manner. Indeed, analogous amino acid substitutions of these non-conserved residues led to distinct extents of gain- (GoF) or loss-of-function (LoF). Moreover, we showed that enhanced overall hydrophobicity along TM3 correlates with an increase in GoF mutant currents. Conclusively, while the overall activation mechanisms of Orai channels appear comparable, there are considerable variations in gating checkpoints crucial for pore opening. The elucidation of regions responsible for isoform-specific functional differences provides valuable targets for drug development selective for one of the three Orai homologs.

## Graphical Abstract

Orai1 and Orai3 channel activation depends in an isoform-specific manner on two non-conserved residues in TM3 (Orai1: V181, L185, Orai3: A156, F160). Mutation of these residues to alanine leads in the absence of STIM1 to small constitutive activity of the respective Orai1 mutants, however, to huge constitutive currents of the respective Orai3 mutants. Overall, two non-conserved residues in TM3 control the maintenance of the closed state as well as an opening permissive conformation of Orai channels in an isoform-specific manner.

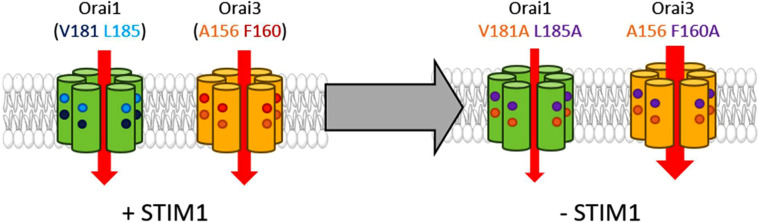

## Introduction

A plethora of processes in the human body, for instance, the immune system or neuronal signaling, is triggered by elevations of cytosolic calcium (Ca^2+^) ion concentrations (Berridge et al., 2000, 2003; Lee et al., 2010). The Ca^2+^ release-activated Ca^2+^ (CRAC) channel represents a major pathway for Ca^2+^ transport into the cell (Feske et al., 2006; Parekh, 2008). It is activated upon the release of intracellular Ca^2+^ from the endoplasmic reticulum (ER). The link between Ca^2+^ store depletion and subsequent Ca^2+^ entry from the extracellular space into the cell is fully established by two proteins, the stromal interaction molecule 1 (STIM1) and Orai. While STIM1 is a Ca^2+^ sensing protein in the ER membrane, Orai proteins are highly Ca^2+^ selective ion channels located in the plasma membrane (PM) (Liou et al., 2005; Zhang et al., 2005, 2006; Feske et al., 2006; Mercer et al., 2006; Peinelt et al., 2006; Prakriya et al., 2006; Soboloff et al., 2006; Spassova et al., 2006; Vig et al., 2006; Wu et al., 2006; Yeromin et al., 2006; Cahalan and Chandy, 2009). ER Ca^2+^ depletion initiated via receptor-ligand binding at the plasma membrane leads to STIM1 activation, its coupling to and activation of Orai channels (Wu et al., 2006; Barr et al., 2008; Muik et al., 2008; Cahalan, 2009; Calloway et al., 2009; Park et al., 2009; Muik et al., 2011; Srikanth and Gwack, 2013; Zhou et al., 2013; Gudlur et al., 2014; Butorac et al., 2020). CRAC channels are highly Ca^2+^ selective with currents exhibiting a reversal potential in the range of +50 mV. Typical CRAC channel hallmarks further include fast Ca^2+^ dependent inactivation (FCDI), enhancement in currents in a divalent free (DVF) Na^+^- compared to a Ca^2+^- containing solution and inhibition by 50 μM 2-APB (Prakriya and Lewis, 2006; Yamashita et al., 2007; Derler et al., 2018; Krizova et al., 2019).

Several structures of *Drosophila melanogaster* Orai (dOrai), two in the closed state and four of constitutively open dOrai mutants, are currently available (Hou et al., 2012, 2018; Liu et al., 2019; Hou et al., 2020). These structures consistently suggest that Orai channels form hexameric complexes. Each subunit is composed of four transmembrane domains that are connected by two extracellular loop regions and a cytosolic one, and flanked by an N- and a C-terminal strand (Feske et al., 2006; Hewavitharana et al., 2007; Hou et al., 2012). The pore region in the center of the channel complex is established by six TM1 domains and is surrounded by TM2 and TM3 in a second and by TM4 in a third ring (Zhou et al., 2016, 2017). At the end of TM4, a bent region, the so-called nexus, forms the connection to the C-terminus. Orai C-termini represent the main coupling sites for STIM1 (Li et al., 2007; Muik et al., 2008; Park et al., 2009; Derler et al., 2013; McNally et al., 2013; Zheng et al., 2013; Palty and Isacoff, 2015). Other cytosolic regions are also essential for STIM1 mediated activation (Derler et al., 2013, 2018; Fahrner et al., 2018; Butorac et al., 2019), however, whether they function as direct interaction sites for STIM1 is still a matter of debate.

An arsenal of Orai1 gain- (GoF) and loss-of-function (LoF) mutants led to the hypothesis that channel activation is accompanied by interdependent TM domain motions (Yeung et al., 2018; Tiffner et al., 2020b, 2021). We recently demonstrated via a screen of double mutants, systematically combining one GoF and one LoF mutation, that Orai1 pore opening indispensably requires global conformational changes of the channel complex and clearance of a series of gating checkpoints (Tiffner et al., 2020b). Structural resolutions together with functional studies suggest that the main conformational changes upon Orai1 activation occur along the pore-lining TM1 and at the outmost side of the channel complex (Hou et al., 2012, 2018, 2020; Liu et al., 2019). While structural alterations within the pore are well understood, the extent of structural changes at the Orai1 channel periphery is still under discussion (Butorac et al., 2020; Zhou et al., 2019). Molecular dynamic (MD) simulations suggest twist-to-open motions with counter-clockwise rotations of TM1 and dilation of the pore at the extracellular side (Dong et al., 2019). At the intracellular side, alternate Orai1 subunits either move outward or show a clockwise rotation. The most recent cryo-EM structure suggests that Orai activation is accompanied by rigid body outward movements of each subunit (Hou et al., 2020).

The Orai protein family consists of three homologs: Orai1, Orai2, and Orai3. Their commonalities include store-operated STIM1 mediated activation, high Ca^2+^ selectivity and overall structural design (Prakriya et al., 2006; Lis et al., 2007). Nevertheless, they possess several distinct functional and structural characteristics.

While TM1 is fully conserved among Orai isoforms, TM2, TM3, and TM4 have approximately 80% sequence identity. Substantial distinctions in the sequence occur in the cytosolic- and extracellular domains (Shuttleworth, 2012; Hogan and Rao, 2015; Fahrner et al., 2018; Krizova et al., 2019). These differences are responsible for a variety of functional alterations.

STIM1 mediated maximum Orai1 currents are 2–3 fold enhanced compared to that of Orai2 and Orai3 (Lis et al., 2007; Frischauf et al., 2009). This difference occurs likely due to the presence of a polybasic- and proline-rich region in the N-terminus of Orai1, but not in that of Orai2 and Orai3 (Li et al., 2007; Takahashi et al., 2007; Fahrner et al., 2009; Yuan et al., 2009). Moreover, FCDI is three times more pronounced for Orai3 compared to that of Orai1 and Orai2. Additionally, only STIM1 mediated Orai1 currents exhibit subsequent to fast inactivation, reaching its maximum within the first 100 ms, a reactivation phase upon the application of a hyperpolarizing voltage step (Lis et al., 2007; Lee et al., 2009; Schindl et al., 2009; Derler et al., 2018; Krizova et al., 2019). The reasons for these isoform-specific inactivation profiles are variations in the cytosolic regions of Orai channels (Lee et al., 2009; Frischauf et al., 2011). Well-known enhancements of CRAC channel currents in a DVF versus a Ca^2+^ containing solution vary for Orai isoforms. The ratio of currents I_*DVF*_:I_*Ca*__2__+_ is lower for STIM1/Orai1 versus STIM1/Orai3, likely due to less pronounced FCDI of Orai1 compared to Orai3 (Prakriya and Lewis, 2006; Derler et al., 2018; Krizova et al., 2019). Moreover, STIM1 mediated currents of Orai isoforms respond distinctly to the well-known drug 2-aminoethyldiphenyl borate (2-APB). Fifty μM 2-APB inhibit STIM1 induced Orai1 and Orai2 currents, while Orai3 currents independent of the presence of STIM1 display strongly enhanced, double rectifying currents (Lis et al., 2007; Derler et al., 2008; Peinelt et al., 2008; Putney, 2010; Krizova et al., 2019). Furthermore, Orai isoforms responded with distinct pharmacological profiles to the two compounds Synta66 and IA65 (Zhang et al., 2020).

Coiled-coil probability predictions revealed that this structural arrangement is 15–17 fold higher for Orai2 and Orai3 C-terminus compared to the Orai1 C-terminus (Frischauf et al., 2009). A single point mutation (L273S/D) within Orai1 C-terminus is sufficient to abolish STIM1 coupling and mediated activation. Contrary, in Orai2 and Orai3 C-termini, two point mutations are required to completely impair STIM1 mediated activation. Analogously, also within STIM1 C-terminus, a single point mutation was sufficient to abolish coupling to Orai1, while a double point mutation was required to block coupling to Orai2 and Orai3 (Li et al., 2007; Muik et al., 2008; Frischauf et al., 2009).

Furthermore, we have recently reported that the communication of the Orai1 N-terminus and the loop2 region is indispensable for Orai1 pore opening and occurs in an isoform-specific manner (Derler et al., 2013). This isoform-specific communication is especially reflected by analog Orai N-terminal truncation mutants, among which only those of Orai1 lose function, while the ones of Orai2 and Orai3 remain functional, despite the remaining N-terminal region is fully conserved. The latter is subject to distinct structural properties of the loop2, the cytosolic portion connecting TM2 and TM3 (Derler et al., 2013; Fahrner et al., 2018). In contrast to Orai3, the flexible loop2 region in Orai1 is longer and thus, forms inhibitory contacts with Orai1 N-terminus as soon as it is truncated to a certain position. Only the swap of Orai3 loop2 restores the activity of the loss-of-function Orai1 N-terminal truncation mutants (Fahrner et al., 2018).

Typically, loss of activity of constitutive Orai1 mutants due to certain N-terminal deletions can be restored by the swap of Orai3-loop2 also in the absence of STIM1 (Fahrner et al., 2018). Intriguingly, this is not the case for the constitutively active Orai1 hinge mutant, containing the substitutions _261_ANSGA_265_ at the bent connection between TM4 and the C-terminus (Zhou et al., 2016). This constitutive channel loses its function upon its N-terminal truncation and remains non-functional also upon the exchange of its loop2 by that of Orai3 (Butorac et al., 2020).

In the present study, we report that Orai TM3 controls the closed and the open state of Orai1 and Orai3 in an isoform-specific manner. This distinct regulation is accomplished by two non-conserved residues in TM3 (in Orai1: V181, L185; in Orai3: A156, F160) which are required for both, the maintenance of the closed state and an opening permissive conformation. Orai isoform-specific differences of TM3 together with the loop2 regions determine the extent of pore opening and Ca^2+^ ion currents.

## Experimental Procedures

### Molecular Biology

For N-terminal fluorescence labeling of human Orai1 (Orai1; accession number NM_032790, provided by the laboratory of A. Rao) as well as human Orai3 (Orai3; accession number NM_152288, provided by the laboratory of L. Birnbaumer), the constructs were cloned into the pEYFP-C1 (Clontech) expression vector via *Kpn*I/*Xba*I (Orai1) and *Bam*HI/*Xba*I (Orai3) restriction sites, respectively. Chimeric constructs were cloned via SOEing (Splicing by Overlap Extension) into the pEYFP-C1 (Clontech) expression vector for N-terminal fluorescence labeling. Site-directed mutagenesis of all the mutants was performed using the QuikChange^TM^ XL site-directed mutagenesis kit (Stratagene) with the corresponding Orai1, Orai3 and/or Orai1-Orai3 chimeric constructs serving as a template, respectively.

Human STIM1 (STIM1; Accession number: NM_003156), N-terminally ECFP-tagged, was kindly provided by T. Meyer’s Lab, Stanford University.

The integrity of all resulting clones was confirmed by sequence analysis (Eurofins Genomics/Microsynth).

### Cell Culture and Transfection

The transient transfection of human embryonic kidney (HEK) 293 cells was performed (Derler et al., 2006) using the TransFectin Lipid Reagent (Bio-Rad) (New England Biolabs). For each transfection, Orai1 plasmids together with STIM1 plasmids were used at a 1:1 ratio, while for Orai3 constructs together with STIM1 plasmids at a 1.5:1 ratio. Regularly, potential cell contamination with mycoplasma species was tested using VenorGem Advanced Mycoplasma Detection kit (VenorGEM).

### Ca^2+^ Fluorescence Measurements

HEK293 cells, transfected with a ratio of 1:1.5 and 1.5:1.5 for the Orai1/R-Geco1.2 (purchased from Addgene, Wu et al., 2013) and Orai3/R-Geco1.2 plasmids, respectively, were grown on coverslips for 1 day. Coverslips were transferred to an extracellular solution without Ca^2+^ and mounted on an Axiovert 135 inverted microscope (Zeiss, Germany) equipped with a sCMOS-Panda digitale Scientific Grade camera 4.2 MPixel and a LedHUB LED Light-Engine light source. Excitation of R-Geco1.2 was obtained using the LED spanning 505 – 600 nm together with a Chroma filter allowing excitation between 540 and 580 nm. Ca^2+^ measurements are shown as normalized intensities of R-Geco1.2 fluorescence in HEK293 cells. Image acquisition and intensity recordings were performed with Visiview5.0.0.0 software (Visitron Systems). A Thomas Wisa perfusion pump was used for extracellular solution exchange during the experiment. All experiments were performed on 3 days and at room temperature.

### Electrophysiology

Electrophysiological recordings that assessed the characteristics of 2–3 constructs were carried out in paired comparison on the same day. Expression patterns and levels of the various constructs were carefully monitored by fluorescence microscopy and were not significantly changed by the introduced mutations. Electrophysiological experiments were performed at 20–24°C, using the patch-clamp technique in the whole-cell recording configuration. For Orai1, Orai3, STIM1/Orai1, STIM1/Orai3 as well as STIM1/Orai1-Orai3 chimera current measurements, voltage ramps were usually applied every 5 s from a holding potential of 0 mV, covering a range of –90 to +90 mV over 1 s. Voltage step protocols were applied from a holding potential of 0 mV to –70 mV for 1.5 s to determine FCDI. The internal pipette solution for passive store-depletion contained (in mM) 3.5 MgCl_2_, 145 Cesium Methane Sulfonate, 8 NaCl, 10 HEPES, 20 EGTA, pH 7.2. Extracellular solution consisted of (in mM) 145 NaCl, 5 CsCl, 1 MgCl_2_, 10 HEPES, 10 glucose, 10 CaCl_2_, pH 7.4. Na^+^-DVF solution contained (in mM) 150 NaCl, 10 HEPES, 10 glucose, and 10 EDTA pH 7.4. Applied voltages were not corrected for the liquid junction potential, which was determined as +12 mV. All currents were leak-corrected by subtraction of the leak current which remained following 10 μM La^3+^ application. All experiments were carried out at least on two different days.

Bar graphs in the figures display for Orai1 proteins in the absence of STIM1 the current density at *t* = 0 s, while in the presence of STIM1 maximum current densities are shown.

### Confocal Fluorescence Microscopy

Confocal microscopy for localization experiments was performed similarly to Singh et al. (2006) and for NFAT subcellular localization studies in analogy to Schober et al. (2019). In brief, a CSU-X1 Real-Time Confocal System (Yokogawa Electric Corporation) was used for recording fluorescence images connected to two CoolSNAP HQ2 CCD cameras (Photometrics) and a dual port adapter (dichroic: 505lp; cyan emission filter: 470/24; yellow emission filter: 535/30; Chroma TechnologyCorp., United States). All these parts were connected to an Axio Observer Z1 inverted microscope (Carl Zeiss) with two diode lasers (445 and 515 nm, Visitron Systems) and placed on a Vision IsoStation anti-vibration table (Newport Corporation). The VisiView software package (v2.1.4, Visitron Systems) was used for controlling and image generation of the confocal system. Threshold determination and background subtraction for image correction had to be done. YFP and CFP images were recorded with an illumination-time of about 300–400 ms.

Images of Orai isoforms as well as NFAT localization were created and analyzed with a custom-made software integrated into MATLAB (v7.11.0, The MathWorks, Inc.). ImageJ was employed for subcellular localization analysis of the NFAT transcription factors by intensity measurements of the cytosol and nucleus, distinguishing between three different populations with different nucleus/cytosol ratios: inactive (<0.85), homogenous (0.85–1.15), and active (>1.15).

All experiments were performed on three different days at room temperature and the resulting data are presented as mean ± S.E.M. (standard error of the mean) for the indicated number of experiments.

### Hydrophobicity Profiles

Hydrophobicity profiles (Rose et al., 1985) were determined with a window size of nine amino acids^1^.

### Statistics

Results are presented as means ± S.E.M. calculated for the indicated number of experiments. The Mann–Whitney test was performed for statistical comparison of two independent samples considering differences statistically significant at *p* < 0.05. For multiple independent samples, we tested for variance homogeneity by Levene Test. As variance homogeneity was not fulfilled, we performed instead of the ANOVA test, the Welch-ANOVA test. Subsequent to Welch-ANOVA we performed the Games-Howell *post hoc* test to determine the pairs which differ statistically significant (*p* < 0.05). Shapiro–Wilk-Test was applied to prove the normal distribution of the respective datasets.

## Results

### TM3 Maintains the Closed State of Orai1 and Orai3 in an Isoform-Specific Manner

**FIGURE 1:**
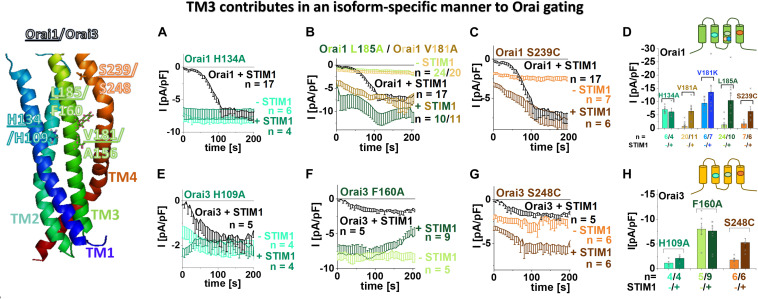
TM3 contributes in an isoform-specific manner to Orai gating. Scheme of Orai1 subunit highlights the positions of the residues within Orai1 and Orai3 channels essential in maintaining the closed state and controlling an opening permissive conformation as tested in **(A–H)**. **(A–C)** Time courses of current densities after whole-cell break-in of Orai1 H134A, Orai1 V181A, Orai1 L185A, Orai1 S239C in the absence and presence of STIM1 in comparison to STIM1 + Orai1 wild-type. **(D)** Block diagram of whole-cell current densities recorded in **(A–C)** and in addition Orai1 V181K in the absence and presence of STIM1. **(E–G)** Time courses of current densities after whole-cell break-in of Orai3 H109A, Orai3 F160A, Orai3 S248C [representing Orai3 analogous mutations to those of Orai1 (H134A, L185A, S239C) recorded in **(A–C)**] in the absence and presence of STIM1 in comparison to STIM1 + Orai3 wild-type. **(H)** Block diagram of whole-cell current densities recorded in **(E–G)**. Mann–Whitney test was employed for statistical analyses with differences considered statistically significant at *p* < 0.05 with a special focus on constitutive Orai TM3 mutants. Constitutive currents of Orai3 F160A are statistically significantly different compared to those of Orai1 V181A as well as Orai1 L185A, respectively.

Recently, two independent site-directed mutagenesis screens on Orai1 TM domains revealed more than a dozen GoF mutations, suggesting that these positions are critical to maintain the closed state (Yeung et al., 2018; Tiffner et al., 2020b). Comparison of these residues (Yeung et al., 2018; Tiffner et al., 2020b) with analog positions in Orai3 TM domains revealed that most of them are fully conserved, except two positions in TM3 (Table 1), which are explained in the paragraph after the next. Initially, we investigated a few conserved positions in TM2 and TM4. In TM2, the single point mutant Orai3 H109A exhibits constitutive activity, in analogy to the GoF-mutant Orai1 H134A (Frischauf et al., 2017). Both, Orai1 H134A and Orai3 H109A reached in the absence and presence of STIM1 maximal current levels similar to those of STIM1 mediated Orai1 or Orai3 currents (Figures 1A,D,E,H). In TM4, Orai3 S248C (analog of the GoF mutant Orai1 S239C) (Figures 1C,D,G,H) and Orai3 P254L (analog of the GoF mutant Orai1 P245L) (Nesin et al., 2014; Derler et al., 2018) exhibit also constitutive activity. Constitutive currents of both, Orai1 S239C as well as Orai3 S248C in the absence of STIM1 are further enhanced in the presence of STIM1 (Figures 1C–E,G,H).

**TABLE 1:** Comparison of analog positions in Orai1 and Orai3 known especially from Orai1 to be involved in the maintenance of the closed state and formation of an opening-permissive pore conformation (Yeung et al., 2018; Tiffner et al., 2020b).

TM1		TM2		TM3		TM4	
Orai1	Orai3	Orai1	Orai3	Orai1	Orai3	Orai1	Orai3
V107	V82	L130	L105	E190	E165	*A235*	*Q243*
V102	V77	H134	H109	F187	F162	S239	S248
F99	F74	F136	F111	***L185***	***F160***	P245	P254
G98	G73	A137	A112	***V181***	***A156***	F250	F259
S97	S72	L138	L113	W176	W151		
		S141	S116				

*The main focus of the study lied on bold-marked non-conserved residues of TM3 domain.*

Moreover, we recently discovered that among the residues which contribute to the maintenance of the closed state, some additionally determine an opening permissive channel conformation. This was proven via site-directed mutagenesis leading at a single position in dependence of the inserted amino acid not only to gain- but also to loss-of-function (GoF and LoF, respectively) (Frischauf et al., 2017; Yeung et al., 2018; Tiffner et al., 2020b). In analogy to Orai1 LoF mutants (Orai1 H134W, Orai1 S239W), we discovered that Orai3 H109W and Orai3 S248W are LoF mutations as well (Supplementary Figures 1a,c,d).

In TM3, the non-conserved residues that maintain the closed state, represent V181 and L185 in Orai1 which correspond to A156 and F160 in Orai3 (Figure 1, scheme and Table 1). Both single point mutants, Orai1 V181A and Orai1 L185A, led to small constitutive activity in the absence of STIM1, which is further enhanced in the presence of STIM1 (Figures 1B,D). Despite the V181A mutation mimics the analog position A156 in Orai3 wild-type, Orai3 maintains the quiescent state. This position will be focused on later in the text. Interestingly, the analog mutant of Orai1 L185A, Orai3 F160A, displays significantly enhanced constitutive activity (Figures 1F,H) to similar extents in the absence and presence of STIM1 in comparison to STIM1 mediated Orai3 wild-type currents. Due to the distinct extents of constitutive currents upon alanine substitutions at these non-conserved positions in TM3, we suppose that they contribute to the maintenance of the closed state in an isoform-specific manner.

Diverse other mutations of F160 led either to gain-of-function or STIM1-mediated activation (Supplementary Figures 1h–j), in analogy to Orai1 L185X mutants (Tiffner et al., 2020b). While Orai3 F160W showed only store-operated activation in the presence of STIM1, Orai3 F160L displayed tiny constitutive currents, which were further enhanced in the presence of STIM1. Orai3 F160S and Orai3 F160G exhibit constitutive activity similar to Orai3 F160A (Supplementary Figures 1h–j). We discovered no mutation of F160 in Orai3 that led to loss-of-function similar to our findings for L185 in Orai1 (Tiffner et al., 2020b).

Among different amino acid substitutions at position A156 in Orai3, it is of note that Orai3 A156K led to robust constitutive activity (Supplementary Figures 1e,g) similar to its analog Orai1 V181K (Figure 1D and Tiffner et al., 2020b). Orai3 A156G, Orai3 A156L and Orai3 A156S exhibited activation only in a store-operated manner via STIM1. Orai3 A156W is a LoF-mutant in the absence as well as the presence of STIM1 (Supplementary Figures 1e–g), in contrast to its analog Orai1 V181W which activated in a store-operated manner by STIM1 (Tiffner et al., 2020b). Orai3 A156F, interestingly, lost plasma membrane expression (Supplementary Figure 2a), contrary to its analog Orai1 V181F, representing a LoF mutant (Tiffner et al., 2020b) with maintained plasma membrane expression (Supplementary Figure 2b). Thus, position V181 in Orai1 and its analog A156 in Orai3 are further involved in an isoform-specific manner in the formation of an opening-permissive conformation.

To determine whether other conserved hydrophobic residues possess also an isoform-specific role on Orai function, we investigated additionally the impact of some of them along TM3, in particular, one helical turn up- (Orai1: F178; Orai3 F153) or downstream (Orai1:L188; Orai3: L163) to the two non-conserved residues (Supplementary Figure 2, schemes). However, only the substitution of the non-conserved residues to serine varied in their impact on activation, while the mutation of the conserved residues to serine led to a comparable behavior in both Orai isoforms (Supplementary Figures 2c,d).

In summary, we discovered that two non-conserved positions in TM3 contribute to a distinct extent to the maintenance of the closed state of Orai channels. Indeed, diverse amino acid substitutions at the analog positions L185 in Orai1 and F160 in Orai3 led to a significant difference in constitutive currents. Additionally, in particular, V181 in Orai1 and A156 in Orai3 configure an opening permissive pore geometry in an isoform-specific manner. In fact, among diverse amino acid substitutions, only the insertion of phenylalanine at V181 in Orai1 and tryptophan at A156 in Orai3 led to loss-of-function.

### Non-conserved Residues in TM3 Modulate Orai1 and Orai3 Mediated Ca^2+^ Entry and NFAT Translocation in an Isoform-Specific Manner

To corroborate our electrophysiological data on the strong difference in the extent of current densities of constitutively active Orai TM3 point mutations, we additionally examined the prominent ones via the complementary assays Ca^2+^ imaging and activation of the transcription factor NFAT (nuclear factor of activated T cells).

**FIGURE 2:**
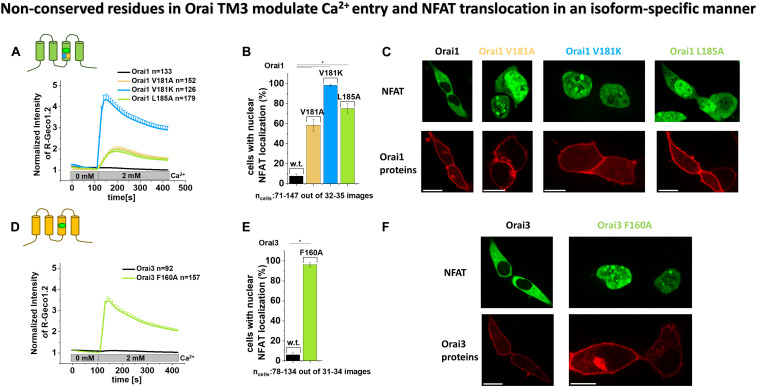
Non-conserved residues in Orai TM3 modulate Ca^2+^ entry and NFAT translocation in an isoform-specific manner. **(A,D)** Cytosolic Ca^2+^ concentrations represented by the normalized intensity of overexpressed R-Geco1.2 were monitored initially in a nominally Ca^2+^ free extracellular solution, followed by a solution containing 2 mM Ca^2+^ in HEK293 cells overexpressing Orai1, Orai1 V181A, Orai1 V181K or Orai1 L185A **(A)** or Orai3 and Orai3 F160A **(D)**. **(B,E)** The average number of HEK293 cells that exhibit nuclear NFAT localization determined upon co-expression (NFAT-CFP) with Orai1 **(B)** or Orai3 **(E)** or corresponding mutants shown in **(A,D)** after 24 h in 2 mM Ca^2+^ containing media. For the analysis 31–34 images of cells containing in total 78–134 cells were used. **(C,F)** Representative images of HEK293 cells co-expressing Orai1 **(C)** or Orai3 **(F)** and corresponding mutants shown in **(A,B,D,E)**, respectively, with NFAT-CFP in the presence of 2 mM Ca^2+^ (Scale bar, 10 μm). In **(A,B)** Welch-ANOVA test (due to lack of variance homogeneity as determined by Levene Test) was used for statistical comparison of Orai1 mutants using the F-distribution *F*(3,252.24) = 98.15, *p* < 0.001 **(A)**; [*F*(3,64.77) = 498.75, *p* < 0.001 **(B)**]. Subsequent to Welch-ANOVA we performed the Games-Howell *post hoc* test to determine the pairs which differ statistically significant (*p* < 0.05). Statistical significance was determined for Orai1 compared to all Orai1 GoF mutants as well as Orai1 V181A and Orai1 L185A compared to Orai1 V181K. In **(D,E)** Mann–Whitney test was employed for statistical analyses with differences considered statistically significant at *p* < 0.05. Statistical significance was determined for Orai3 compared to Orai3 F160A.

As expected, overexpression of Orai1 and Orai3, respectively, in HEK 293 cells yielded no enhancements in Ca^2+^ levels upon the exchange from a 0 mM Ca^2+^ to a 2 mM Ca^2+^ containing solution, as determined via fluorescence intensity of R-Geco1.2 (Figures 2A,D). In contrast, all point mutants Orai1 V181A, Orai1 V181K, Orai1 L185A as well as Orai3 F160A exhibited robust enhancements in Ca^2+^ levels upon the switch to a Ca^2+^ containing solution (Figures 2A,D). Remarkably, in accord with our electrophysiological results, Orai1 V181A and Orai1 L185A showed significantly lower Ca^2+^ entry compared to Orai1 V181K and Orai3 F160A (Figures 2A,D).

Furthermore, while Orai1 and Orai3 expressing cells showed no detectable NFAT translocation to the nucleus, the expression of Orai1 V181A and Orai1 L185A led to NFAT translocation in ∼ 60 – 70% of the cells, and the presence of Orai1 V181K and Orai3 F160A triggered NFAT translocation in ∼ 90 -100% of the cells (Figures 2B,C,E,F). These results are in accordance with the enhanced constitutive activity of Orai1 V181K and Orai3 F160A versus Orai1 V181A and Orai1 L185A (Figures 1B,D,F,H, 2A,D).

In agreement with our electrophysiological evidence, both, our Ca^2+^ imaging and NFAT translocation results, reflect the distinct activities of analog constitutive Orai1 and Orai3 point mutants. Alanine substitutions of the non-conserved residues V181 and L185 in TM3 cause only low constitutive activity. Contrary, A156 in Orai3 wild-type (analog to V181A in Orai1) maintains the closed state, while the F160A in Orai3 (analog to L185A in Orai1) induces robust constitutive activity to comparable levels as obtained with Orai1 V181K.

### Orai3 Requires Comparable Global TM Domain Motions to Enable Pore Opening as Orai1

We recently showed via a library of Orai1 double mutants, containing each one GoF and one LoF mutation that the LoF mutation acts dominant over the GoF mutations independent of their location relative to each other (Tiffner et al., 2020b). These findings suggested that Orai1 pore opening predominantly requires clearance of a set of checkpoints in and a global conformational change of all TM domains. Here we examined whether this also applies to Orai3 since TM3 maintains the closed state of Orai variants in an isoform-specific manner. To address the latter, we tested several double mutants, each combining a LoF and a GoF mutation, one of which is closer to TM1 than the second within the middle and cytosolic extended transmembrane regions that we previously termed MTR and CETR (Tiffner et al., 2020b).

**FIGURE 3:**
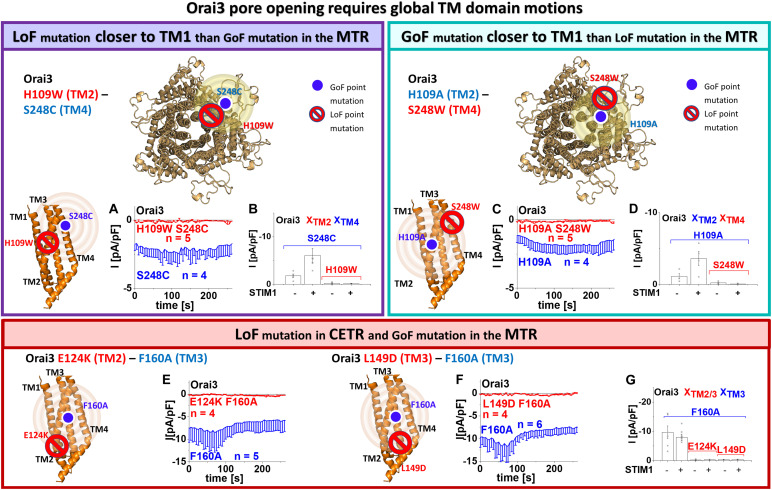
Orai3 pore opening requires global TM domain motions. Schemes representing the location of the investigated residues within a single subunit (top – left, bottom left and middle) of Orai3 or the whole channel complex (top – middle), for either LoF or GoF mutation closer to the pore, respectively. The red stop sign indicates the position of the LoF mutation, while the blue circle shows the position of the GoF mutation. The yellow spheres indicate the impact of the GoF mutation on the entire subunit. Special focus was addressed to H109, F160, and S248, due to their location in TM2, thus, close to the pore, in TM3, thus, in the center of the channel complex or TM4, thus, at the periphery of the channel complex, respectively **(A–D)**. Via combining a GoF and a LoF mutation at the respective position and investigating their impact on each other, we examined whether interdependent TM domain motions within the entire MTR are necessary for pore opening. **(A)** Time courses of current densities after whole-cell break-in of Orai3 S248C compared to Orai3 H109W S248C in the absence of STIM1. Constitutive currents of the MTR-GoF Orai3 S248C mutant are abolished by the additional introduction of the LoF mutation H109W. **(B)** Block diagram of whole-cell current densities of Orai3 S248C, Orai3 H109W S248C in the absence (*t* = 0 s) and the presence (maximum current densities) of STIM1 (*n* = 4–5 cells; values are mean ± SEM). **(C)** Time courses of current densities after whole-cell break-in of Orai3 H109A compared to Orai3 H109A S248W in the absence of STIM1. Constitutive currents of the MTR-GoF Orai3 H109A mutant are inhibited by the additional introduction of the LoF mutation S248W. **(D)** Block diagram of whole-cell current densities of Orai3 H109A, Orai3 H109A S248W in the absence (*t* = 0 s) and the presence (maximum current densities) of STIM1 (*n* = 4 – 5 cells; values are mean ± SEM). Next, special focus was addressed to the LoF mutations E124K, L149D in the CETR **(E–G)**. Via combining a GoF in the MTR and a LoF mutation in the CETR at those respective positions and investigating their impact on each other, we examined whether interdependent TM domain motions within the entire channel complex are necessary for pore opening. **(E,F)** Time courses of current densities after whole-cell break-in of Orai3 F160A compared to Orai3 E124K F160A in the absence of STIM1 and Orai3 F160A compared to Orai3 L149D F160A, respectively. Constitutive currents of the MTR-GoF Orai3 F160A mutant are abolished by the additional introduction of the LoF mutation E124K or L149D. **(G)** Block diagram of whole-cell current densities of Orai3 F160A, Orai3 E124K F160A, Orai3 L149D F160A in the absence (*t* = 0 s) and the presence (maximum current densities) of STIM1 (*n* = 4 – 6 cells; values are mean ± SEM). The Welch-ANOVA test (due to lack of variance homogeneity as determined by Levene Test) was used for statistical comparison of Orai3 mutants in **(B,D,G)** using the F-distribution [*F*(3,9.19) = 12.91, *p* < 0.005 **(B)**, *F*(3,5.53) = 17.86, *p* < 0.005 **(D)**, *F*(5,10.69) = 29.15, *p* < 0.001 **(G)**]. Subsequent to Welch-ANOVA we performed the Games-Howell *post hoc* test to determine the pairs which differ statistically significant (*p* < 0.05). Statistical significance was determined for Orai3 GoF mutant currents compared to Orai3 GoF-LoF double mutant currents **(B,D,G)**.

Initially, analogous to what we have shown previously in Tiffner et al. (2020b), we investigated an Orai3 double point mutant combining a LoF mutation in TM2, H109W, closer to TM1 with a GoF mutation in TM4, S248C, thus, farther apart from TM1, both within the MTR. As expected, we discovered that the H109W acts dominant over S248C (Figures 3A,B). Similarly, Orai3 H109W P254L, also with the LoF mutation closer to TM1 than the GoF mutation, exhibited loss of function (Supplementary Figure 3a).

Next, we investigated an Orai3 double point mutant with the GoF mutation (H109A in TM2) closer to the pore than the LoF mutation (S248W in TM4) within the MTR. In analogy to our findings with Orai1 (Tiffner et al., 2020b), we discovered loss-of-function for Orai3 H109A S248W (Figures 3C,D). Hence, the LoF mutations act dominant over the respective GoF mutations, independent of their location relative to each other and the pore.

Moreover, we investigated two other Orai3 LoF mutations in the CETR of TM2 and TM3, E124K (analog to E149K in Orai1) and L149D (analog to L174D in Orai1) (Supplementary Figure 3b) for their effect on the constitutively active Orai3 F160A (Figures 3E–G). Both LoF mutations act dominant over the GoF mutation, as shown via the impaired activity of Orai3 E124K F160A and Orai3 L149D F160A.

Overall, despite Orai3-TM3 exhibits distinct features than that of Orai1 in maintaining the closed state of the channel, both isoforms require a global conformational change of the channel complex to establish pore opening.

### Swapping Non-conserved Residues in TM3 Unravels Their Significance in Orai Channel Gating

**FIGURE 4:**
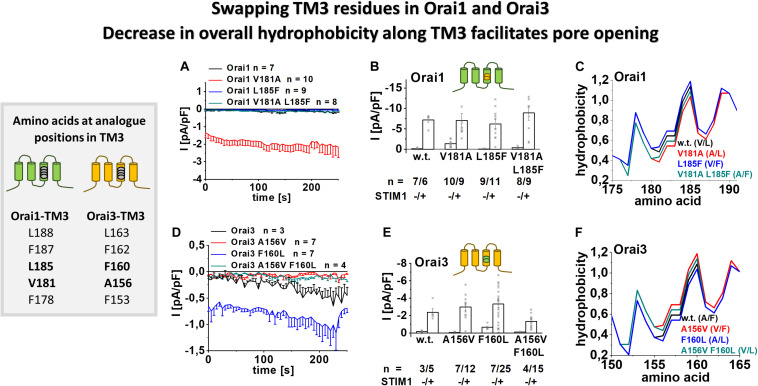
Swapping TM3 residues in Orai1 and Orai3 shows that a decrease in overall hydrophobicity along TM3 facilitates pore opening. Table depicting amino acids at the analog position in TM3 in Orai1 and Orai3 (left). **(A)** Time courses of current densities after whole-cell break-in of Orai1 V181A, Orai1 L185F and Orai1 V181A L185F (thus mimicking the positions A156 and F160 in Orai3) compared to Orai1 in the absence of STIM1. **(B)** Block diagram of whole-cell current densities of Orai1, Orai1 V181A, Orai1 L185F, and Orai1 V181A L185F in the absence (*t* = 0 s) and the presence (maximum current densities) of STIM1 (*n* = 6–11 cells; values are mean ± SEM). **(C)** Hydrophobicity plots of the Orai1 TM3 region (amino acid 175–190) for mutants investigated in **(A)**. **(D)** Time courses of current densities after whole-cell break-in of Orai3 A156V, Orai3 F160L and Orai3 A156V F160L (thus mimicking the positions V181 and L185 in Orai1) compared to Orai3 in the absence of STIM1. **(E)** Block diagram of whole-cell current densities of Orai3, Orai3 A156V, Orai3 F160L, and Orai3 A156V F160L in the absence (*t* = 0 s) and the presence (maximum current densities) of STIM1 (*n* = 3–25 cells; values are mean ± SEM). **(F)** Hydrophobicity plots of the Orai3 TM3 region (amino acid 150–165) for mutants investigated in **(D)**. Mann–Whitney test was employed for statistical analyses with differences considered statistically significant at *p* < 0.05. Statistical significance was determined for different Orai channel currents, either in the absence or presence of STIM1 via pairwise comparison. Currents of Orai1 wild-type., Orai1 L185F and Orai1 V181A L185F are statistically significantly different to those of Orai1 V181A **(B)** in the absence of STIM1. Currents of Orai3 wild-type, Orai3 A156V and Orai3 A156V F160L are statistically significantly different to those of Orai3 F160L **(E)** in the absence of STIM1.

To investigate whether mainly the non-conserved residues in TM3 are responsible for the isoform-specific maintenance of the closed state, we swapped the respective residues in Orai1 and Orai3 (Figure 4, table). Orai1 V181A, thus mimicking Orai3 A156, is constitutively active. In contrast, Orai1 L185F, thus analog to Orai3 F160, and Orai1 V181A L185F, thus representing TM3 in particular in terms of A156 and F160 in wild-type Orai3, remain both inactive in the absence of STIM1 (Figures 4A,B). Vice versa, Orai3 shows constitutive activity upon the F160L mutation, thus mimicking Orai1 at this site. In contrast, Orai3 A156V, which mimics Orai1 V181, and Orai3 A156V F160L, which mimics both V181 and L185 in Orai1, remain inactive in the absence of STIM1 (Figures 4D,E). This indicates that distinct side-chain properties at analog positions in TM3 of Orai1 and Orai3 determine isoform-specific maintenance of the closed and open state. In the presence of STIM1, all mutants show store-operated activation typical for CRAC channels (Figures 4B,E and Supplementary Figures 3c,d).

Moreover, we performed the bioinformatic analysis by hydrophobicity profiles, particularly along TM3, employing a prediction program (see section “Materials and Methods”) based on the Rose and Lesser hydrophobicity scale. The hydrophobicity profile of Orai1 and Orai3 displayed four transmembrane domains in correlation with recent studies (Derler et al., 2009) (Supplementary Figures 3e,f). The mutation of the valine to alanine in Orai1 (at position 181) caused a reduction in overall hydrophobicity along TM3. In contrast, the introduction of the single point mutation L185F or the double mutations V181A L185F caused an increase in overall hydrophobicity along TM3 (Figure 4C). In Orai3, the mutation F160L led to a reduction, while A156V and A156V F160L caused an enhancement in the overall hydrophobicity along TM3 (Figure 4F). Hence, the two constitutively active Orai mutants, Orai1 V181A and Orai3 F160L, displayed the lowest overall hydrophobicity along TM3 (Figures 4C,F). In contrast, other mutants only active upon store-dependent, STIM1-mediated activation, display an overall hydrophobicity along TM3 comparable or even higher than that of wild-type Orai proteins.

Summarizing, we discovered that the constitutive activity of certain Orai mutants, thus, together with maintenance of the closed state of Orai channels in resting conditions, correlates with the overall hydrophobicity along TM3.

### Enhanced Hydrophobicity at A156 in Orai3 Reduces the Constitutive Activity of Orai3 F160A

**FIGURE 5:**
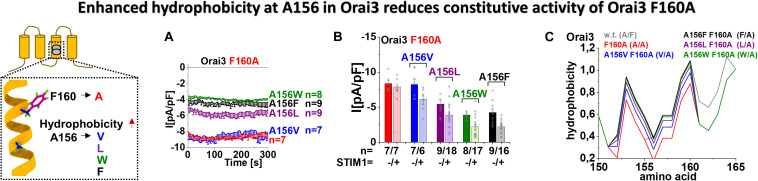
Enhanced hydrophobicity at A156 in Orai3 reduces the constitutive activity of Orai3 F160A. The scheme represents Orai3 with a special focus on the two non-conserved residues in TM3 (F160 and A156). Those positions are examined by mutations at position A156 toward different hydrophobic amino acids within the background of the constitutive mutant F160A. **(A)** Time courses of constitutive current densities after whole-cell break-in of Orai3 F160A, Orai3 A156V F160A, Orai3 A156L F160A, Orai3 A156W F160A, and Orai3 A156F F160A in the absence of STIM1. **(B)** Block diagram of whole-cell current densities of Orai3 mutants tested in **(A)** in the absence (*t* = 0 s) and the presence (maximum current densities) of STIM1 (*n* = 6–18 cells; values are mean ± SEM). **(C)** Hydrophobicity plots showing the Orai3 TM3 region (amino acid 150–165) for mutants investigated in **(A)** compared to Orai3 wild-type. In **(B)** the Welch-ANOVA test (due to lack of variance homogeneity as determined by Levene Test) was used for statistical comparison of Orai3 mutants using the F-distribution [*F*(9,30.65) = 31.14, *p* < 0.001]. After Welch-ANOVA we performed the Games-Howell *post hoc* test to determine the pairs which differ statistically significant (*p* < 0.05). Statistical significance was determined for Orai3 A156L F160A, Orai3 A156W F160A, and Orai3 A156F F160A compared to Orai3 F160A, both in the absence and presence of STIM1.

In case overall hydrophobicity along TM3 determines the extent of constitutive activity, we hypothesized that enhanced hydrophobicity at position A156 in Orai3 decreases the robust, constitutive activity of Orai3 F160A. Thus, we substituted A156 in Orai3 F160A by different amino acids with an enhancing degree of hydrophobicity, in particular, by valine, leucine, phenylalanine, and tryptophan (Figure 5, scheme). Substitution to valine left constitutive activity of the respective double mutant, Orai3 A156V F160A, almost comparable to that of Orai3 F160A. Remarkably, Orai3 A156L F160A, Orai3 A156F F160A and Orai3 A156W F160A led with increased hydrophobicity to a decrease in constitutive activity by about 40 – 50% (Figures 5A,B). Intriguingly, in the presence of STIM1, all Orai3 double mutant currents were reduced compared to those in the absence of STIM1. Nevertheless, similar as in the absence of STIM1, we observed also in the presence of STIM1 with increased hydrophobicity and side-chain size at position A156 a reduction in Orai3 double mutant compared to Orai3 F160A currents (Figure 5B).

Determination of the overall hydrophobicity along TM3 revealed the lowest level for Orai3 F160A in line with its robust constitutive activity. The exchange of A156 by amino acids with enhanced hydrophobicity, as tested in Figures 5A,B, indeed revealed a gradual increase of overall hydrophobicity along TM3 (Figure 5C), in accord with the decrease in currents.

Altogether, we discovered that overall enhancement in hydrophobicity along TM3 in dependence of the inserted amino acid at position 156 in Orai3 correlates with a decrease in constitutive activity of Orai3 F160A. Apparently, Orai3 double mutants did not lose constitutive activity, likely due to the strong impact of the F160A mutation on Orai3 pore opening.

### The Isoform-Specific Role of TM3 Is Supported by the Cytosolic Loop2 Region

**FIGURE 6:**
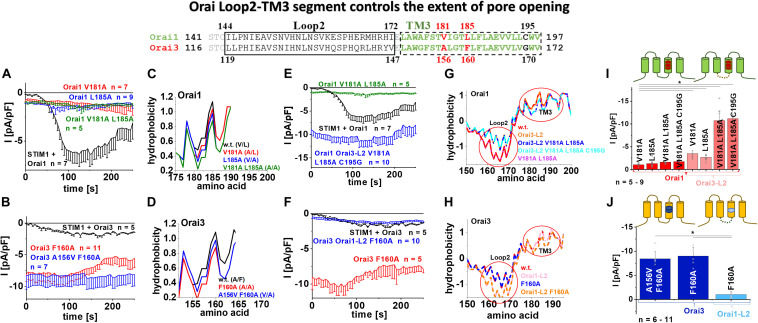
Orai loop2-TM3 segment controls the extent of pore opening. The scheme represents the sequence alignment of Orai1 and Orai3 at the loop2 and TM3 region. The sequence in TM3 is almost fully conserved except three residues highlighted in red and black. **(A)** Time courses of current densities after whole-cell break-in of Orai1 V181A, Orai1 L185A and Orai1 V181A L185A in the absence of STIM1 compared to Orai1 in the presence of STIM1. **(B)** Time courses of current densities after whole-cell break-in of Orai3 F160A and Orai3 A156V F160A in the absence of STIM1 compared to Orai3 in the presence of STIM1. **(C)** Hydrophobicity plots of the Orai1 TM3 region (amino acid 175–190) for mutants investigated in **(A)** compared to Orai1 wild-type. **(D)** Hydrophobicity plots of the Orai3 TM3 region (amino acid 150–165) for Orai3 F160A and Orai3 A156V F160A compared to Orai3 wild-type. **(E)** Time courses of current densities after whole-cell break-in of Orai1 Orai3-L2 V181A L185A C195G and Orai1 V181A L185A in the absence of STIM1 compared to Orai1 in the presence of STIM1. **(F)** Time courses of current densities after whole-cell break-in of Orai3 Orai1-L2 F160A and Orai3 F160A in the absence of STIM1 compared to Orai3 in the presence of STIM1. **(G)** Hydrophobicity plots of the Orai1-L2-TM3 region (amino acid 150–200) for Orai1 Orai3-L2, Orai1 Orai3-L2 V181A L185A, Orai1 Orai3-L2 V181A L185A C195G, and Orai1 V181A L185A compared to Orai1 wild-type. Loop2 and TM3 are highlighted by red circles. **(H)** Hydrophobicity plots of the Orai3-L2-TM3 region (amino acid 150–195) for Orai3 Orai1-L2, Orai3 F160A, and Orai3 Orai1-L2 F160A compared to Orai3 wild-type. **(I)** Block diagram of whole-cell current densities of Orai1 V181A, Orai1 L185A, Orai1 V181A L185A, Orai1 V181A L185A C195G, Orai1-Orai3-L2 V181A, Orai1-Orai3-L2 L185A, Orai1-Orai3-L2 V181A L185A, and Orai1-Orai3-L2 V181A L185A C195G in the absence of STIM1 (*n* = 5–9 cells; values are mean ± SEM). **(J)** Block diagram of whole-cell current densities of Orai3 F160A, Orai3 A156V F160A, and Orai3 Orai1-L2 F160A in the absence of STIM1 (*n* = 6–11 cells; values are mean ± SEM). In **(I,J)** the Welch-ANOVA test (due to lack of variance homogeneity as determined by Levene Test) was used for statistical comparison of Orai1 and Orai3 mutants using the F-distribution [*F*(6,16.86) = 14.41, *p* < 0.001 **(I)**; *F*(7,22.33) = 32.11, *p* < 0.001 **(J)**]. Subsequent to Welch-ANOVA we performed the Games-Howell *post hoc* test to determine the pairs which differ statistically significant (*p* < 0.05). Asterisks (*) indicates statistical significance compared to Orai1 V181A **(I)** or Orai3 Orai1-L2 F160A **(J)**, respectively.

In the following, we further questioned why only Orai3 F160A, but not the analog Orai1 L185A displays huge constitutive current densities compared to STIM1 mediated Orai activation (Figures 1B,F). Intriguingly, a double point mutant Orai1 V181A L185A exhibited only small constitutive activity (Figures 6A,I), despite the side-chain properties at the mutated positions match with those of Orai3 F160A (Figure 4, table). Remarkably, Orai3 A156V F160A remained strongly constitutively active (Figures 5A,B, 6B,J), although these mutated positions in TM3 correspond to the analog ones in Orai1 L185A (Figure 4, table).

Investigation of the overall hydrophobicity of TM3 revealed for all constitutively active mutants a reduced hydrophobicity compared to their respective wild-type Orai proteins. Interestingly, Orai1 V181A L185A and Orai3 F160A reached the lowest levels for the Orai isoforms (Figures 6C,D,G,H). Thus, alterations of overall hydrophobicity along the TM3 region cannot fully explain distinct levels of current densities.

Next, we exchanged the non-conserved cysteine C195 in Orai1 by a glycine, as present at the analog positions in Orai3 (Figure 6, alignment). However, also Orai1 V181A L185A C195G showed small constitutively active currents similar to Orai1 V181A L185A (Figure 6I and Supplementary Figure 4a).

We recently reported isoform-specific functional differences of Orai1 and Orai3 due to distinct structural properties of the loop2 regions connecting TM2 and TM3 [Orai3-loop2 (L2) – aa:119–147, Orai1-L2 – aa:144–172] (Fahrner et al., 2018). By applying this knowledge, we discovered that Orai1 V181A Orai3-L2 and Orai1 L185A Orai3-L2 showed slightly enhanced constitutive activity compared to the single point mutants without the swapped loop2 (Figure 6I and Supplementary Figures 4b,c). Remarkably, an additional exchange of the loop2 of Orai1 by that of Orai3 (thus mimicking Orai3 at this region; see alignment Figure 6) in Orai1 V181A L185A or also Orai1 V181A L185A C195G led to strongly enhanced constitutive currents, in analogy to Orai3 F160A (Figures 6E,I and Supplementary Figures 4d,e). Vice versa, Orai3 F160A containing the loop2 of Orai1 instead of that of Orai3, thus Orai3 Orai1-L2 (aa:144-172) F160A displayed only small constitutive currents similar to Orai1 V181A, Orai1 L185A or Orai1 V181A L185A (Figures 6F,J).

Analysis of the overall hydrophobicity of TM3 of the respective chimeric constructs revealed no difference compared to wild-type constructs. Interestingly, overall hydrophobicity along the loop2 region was distinct (Figures 6G,H, red circles). The huge constitutive activity could be only obtained upon enhanced hydrophobicity along the loop2 region and reduced hydrophobicity along TM3.

Altogether, those results provide indisputable evidence that distinct magnitudes of the constitutive Orai1- and Orai3-TM3 mutant current densities are not only determined by certain residues in TM3 but rather by the entire loop2-TM3 segments. Thus, the loop2 is not only crucial for an interplay with the Orai N-terminus (Fahrner et al., 2018), but also determines together with TM3 isoform-specific Orai activation.

Due to rather drastic modifications of the strongly constitutively active Orai1 Orai3-L2 chimeras (e.g., Orai1 Orai3-L2 V181A L185A C195G), we still investigated whether their biophysical properties, the so-called authentic CRAC channel hallmarks, are comparable to that of Orai3 F160A (Derler et al., 2018). As shown exemplarily for Orai1 Orai3-L2 V181A L185A C195G (Supplementary Figures 4f–h), constitutive currents exhibit a strongly inward-rectifying current/voltage relationships with a reversal potential of ∼ +50 mV, both in the absence and presence of STIM1 (Supplementary Figure 4f). In the absence of STIM1, the I/V relationship exhibited a U-shaped form at very negative potentials between −85 mV and −70 mV, as we discovered also for Orai3 F160A (Derler et al., 2018) (Supplementary Figure 4f). Upon switching from a Ca^2+^-containing to a DVF Na^+^-solution the Orai1 Orai3-L2 V181A L185A C195G currents decreased in the absence of STIM1 and enhanced in the presence of STIM1 (Supplementary Figure 4g), in accord with our previous findings (Derler et al., 2018). Moreover, fast Ca^2+^ dependent inactivation (FCDI) was completely abolished in the absence of STIM1 and instead reversed into a robust potentiation. The presence of STIM1 fully restored FCDI as known for STIM1-Orai1 currents as well as a diversity of other constitutively active mutants (Derler et al., 2018) (Supplementary Figure 4h). Overall, this constitutive Orai1 Orai3-L2 chimeric mutant shows CRAC channel characteristics similar to Orai3 F160A (Derler et al., 2018).

In summary, the maintenance of the closed state of Orai1 and Orai3 is not only governed in an isoform-specific manner by two non-conserved residues in their TM3 domains (Orai1: V181, L185; Orai3: A156, F160), but further controlled by a distinct overall conformation of the loop2-TM3 region.

### Orai1 Orai3-L2 Chimera Exhibits Analog Isoform-Specific Behavior of TM3 Like Orai1 Wild-Type

**FIGURE 7:**
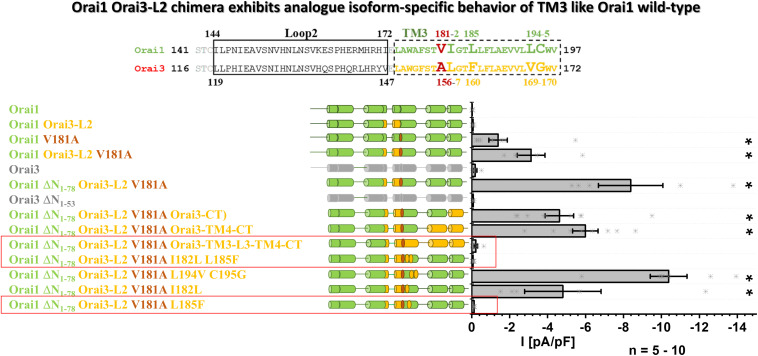
Orai1-Orai3-L2 chimera exhibits analog isoform-specific behavior of TM3 like Orai1 wild-type. The scheme represents the sequence alignment of Orai1 and Orai3 of the loop2 and TM3 region. The sequence in TM3 is almost fully conserved except for residues highlighted by increased letter size. Block diagram of whole-cell current densities of Orai1, Orai1-Orai3-L2, Orai1 V181A, Orai1-Orai3-L2 V181A, Orai3, Orai1-ΔN_1__–__78_ Orai3-L2 V181A, Orai3 ΔN_1__–__53_, Orai1 ΔN_1__–__78_ Orai3-L2 V181A Orai3-CT, Orai1 ΔN_1__–__78_ Orai3-L2 V181A Orai3-TM4-CT, Orai1 ΔN_1__–__78_ Orai3-L2 V181A Orai3-TM3-L3-TM4-CT, Orai1 ΔN_1__–__78_ Orai3-L2 V181A I182L L185F, Orai1 ΔN_1__–__78_ Orai3-L2 V181A L194V C195G, Orai1 ΔN_1__–__78_ Orai3-L2 V181A I182L, and Orai1 ΔN_1__–__78_ Orai3-L2 V181A L185F (*n* = 5–10 cells; values are mean ± SEM) in the absence of STIM1. The schemes highlight the position of V181A mutation and regions of interest typically replaced by those of Orai3. Mann–Whitney test was employed for statistical analyses with differences considered statistically significant at *p* < 0.05. Asterisks (*) indicate statistical significance compared to Orai1 wild-type.

Since Orai1 Orai3-L2 V181A showed enhanced constitutive activity versus Orai1 V181A, we employed a chimeric approach to investigate whether also in this case, only the two non-conserved residues in TM3 make up the isoform-specific control of Orai channel function (Figure 7). To determine whether the different N-termini have an impact on the constitutive activity of Orai1 Orai3-L2 V181A, we deleted the first 78 residues. Nevertheless, this deletion mutant chimera (Orai1 DN_1__–__78_ Orai3-L2 V181A) left constitutive activity unaffected. This is in line with other constitutively active mutants (Derler et al., 2018) and showed that the N-terminal segment 1–78 is not involved in the maintenance of constitutive currents. Next, we tested whether non-conserved residues in TM4 or even the C-terminus influence the constitutive currents of Orai1 DN_1__–__78_ Orai3-L2 V181A. However, neither the swap of Orai1 C-terminal nor Orai1 TM4-C-terminal region by that of Orai3 impaired constitutive activity. Only the exchange of the region starting from TM3 till the end of the C-terminus in Orai1 by that of Orai3 led to the loss of the constitutive activity. This suggests that the sites modulating constitutive activation are located in TM3. Via site-directed mutagenesis of non-conserved residues in TM3 (Figure 7, alignment), we narrowed down the point mutation abolishing constitutive activity of Orai1 DN_1__–__78_ Orai3-L2 V181A. Specifically, we introduced the following point mutations: I182L, L185F, L194V, C195G, thus, exchanging non-conserved residues in Orai1-TM3 by those of Orai3 (Figure 7, alignment). Insertion of the double mutation L194V C195G or the single mutation I182L retained constitutive activity of Orai1 DN_1__–__78_ – Orai3-L2 V181A. Interestingly, insertion of the double mutant I182L L185F (Orai1 DN_1__–__78_ Orai3-L2 V181A I182L L185F) or L185F (Orai1 DN_1__–__78_ Orai3-L2 V181A L185F) led to the loss of constitutive activity of the N-truncated chimera. This finding is in line with the resting state of the analog Orai3 N-truncation mutant, Orai3 DN_1__–__53_ (Figure 7).

In accord with the results in Figure 5, we demonstrate in the background of the Orai3-loop2 that while a V181A substitution in Orai1 can induce constitutive activity, an additional substitution L185F brings Orai1 back into the closed state. This chimeric approach further strengthens our findings that in TM3 predominantly two non-conserved residues, V181 and L185 in Orai1 (A156 and F160 in Orai3), determine the maintenance of the closed state in an isoform-specific manner.

## Discussion

**FIGURE 8:**
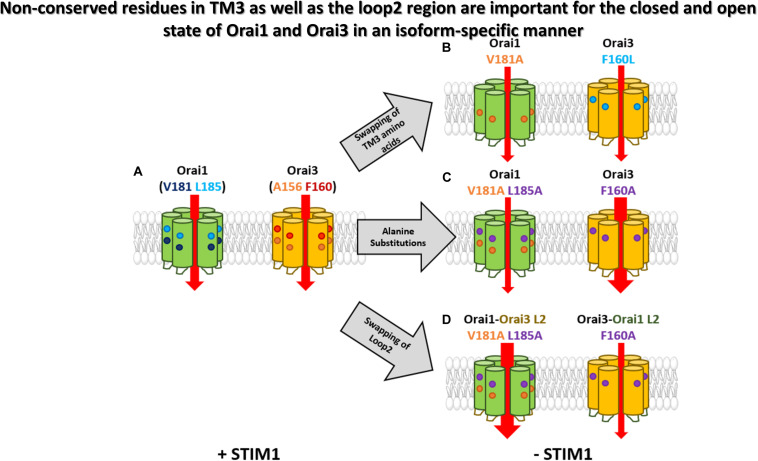
Non-conserved residues in TM3 as well as the loop2 region are involved in the maintenance of the closed state and configure an opening permissive conformation of Orai1 and Orai3 in an isoform-specific manner. A simplified summary of the isoform-specific effects of TM3 of Orai1 and Orai3. **(A)** Orai1 and Orai3 are active in the presence of STIM1. The non-conserved residues in TM3 of each Orai isoform are highlighted. **(B)** Swapping of certain TM3 isoform-specific amino acids (Orai1 V181A and Orai3 F160L) leads to a constitutive activity already in the absence of STIM1. **(C)** Alanine substitutions of non-conserved residues in TM3 reveal only small constitutive currents for Orai1 V181A L185A, while Orai3 F160A displays huge currents even higher than its wild-type analog. **(D)** Additional swapping of the loop2 regions within the mutants of **(C)** also swaps the extent of constitutive activity highlighting the importance of the whole loop2-TM3 region.

In this study, we elucidated an isoform-specific function of Orai gating checkpoints in TM3 (Figure 8). Two non-conserved hydrophobic residues in TM3 contribute to the maintenance of the closed state and the configuration of an opening permissive conformation of Orai1 and Orai3 channels to a different degree, which is in accordance with the overall hydrophobicity along TM3. In addition, the non-conserved Orai loop2 regions contribute to isoform-specific features of Orai channel activation.

We recently demonstrated that Orai1 channel activation requires the clearance of a series of gating checkpoints within all TM domains (Tiffner et al., 2020b). Mutation of the respective checkpoints can lead either to gain- or loss-of-function in dependence of the inserted amino acid. This suggests that the particular residues are involved in both, maintenance of the closed state and the establishment of an opening permissive pore/channel conformation. Here, we showed that most of these checkpoints in TM2, TM3, and TM4 are conserved (Table 1). Only TM3 includes two non-conserved positions (V181, L185 in Orai1 and the analogs A156, F160 in Orai3) (Figure 8A). Analog substitutions of representative gating checkpoints in TM2 and TM4 of Orai1 (Tiffner et al., 2020b) and Orai3 showed comparable extents of gain- or loss-of-function relative to store-operated activation of the wild-type protein. Concerning TM3, mutation of two non-conserved residues to small amino acids, such as alanine or serine, led to distinct magnitudes of current densities, Ca^2+^ entry and NFAT translocation, suggesting that they impact the maintenance of the closed state in an isoform-specific manner (Figure 8C). In addition, only different amino acid substitutions at the analogous positions V181 in Orai1 and A156 in Orai3 led to loss of function (Orai1 V181F, Orai3 A156W). This indicates that this position also influences the establishment of an opening-permissive conformation differently in the respective Orai variants.

Despite these isoform-specific differences in TM3, we provide evidence that both channels necessitate a global conformational change of the channel complex for pore opening. We demonstrated this aspect via several Orai3 double point mutants each containing one LoF and one GoF mutation in the MTR and CETR. In all possible combinations, the LoF mutation acted dominant over the GoF mutation independent of their location relative to each other, leading to overall loss-of-function of Orai3 channels, both, in the absence as well as the presence of STIM1. This is conform with our recent finding on Orai1 (Tiffner et al., 2020b).

Even though global activation mechanisms of Orai channels are identical, a detailed characterization of the individual checkpoints is valuable for potential isoform-specific interferences with Orai channel functions. Our characterization of GoF mutations in TM3 revealed that Orai1 V181A and Orai1 L185A lead to small constitutive activity. Interestingly, despite the analog position of Orai1 V181A in Orai3 contains already an alanine (A156), it remains in the resting state. The reason for the latter is subject to the residue one helical turn downstream which features lower hydrophobicity in Orai1 (L185) than in Orai3 (F160). Indeed, the double mutant Orai1 V181A L185F, thus, mimicking Orai3 at the two positions, lost constitutive activity. Analogously, Orai3 F160L gained constitutive activity, in line with Orai1 V181A (Figure 8B). Thus, in Orai1, both, V181 and L185 contribute to the maintenance of the closed state, while in Orai3, it is predominantly the position F160. Accordingly, overall hydrophobicity along TM3 is enhanced for Orai proteins and mutants retaining the resting state and decreased for Orai mutants showing constitutive activity. Indeed, the robust constitutive activity of Orai3 F160A was reduced upon the substitution of A156 one helical turn upstream by amino acids with enhanced hydrophobicity. Also, the insertion of a strongly hydrophilic lysine at the positions A156 in Orai3 led to robust constitutive currents. Furthermore, the latter finding counteracts the potential argument that an increase in the side-chain size of different hydrophobic amino acids at position A156 in Orai3 F160A decreases constitutive activity.

Remarkably individual substitutions, as well as double point mutations of the two non-conserved residues in Orai1, led to small constitutive activity, while Orai3 F160A exhibited huge constitutive activity compared to maximum currents reached upon STIM1 mediated Orai wild-type activation (Figure 8C). We discovered via the swap of the loop2 region of either Orai1 or Orai3 in either of the constitutively active Orai3 F160A or Orai1 V181A L185A mutants, that currents reduced (Orai3 Orai1-L2 F160A) or enhanced (e.g., Orai1 Orai3-L2 V181A L185A), respectively (Figure 8D). While these chimeras exhibited comparable overall hydrophobicity along TM3, the hydrophobicity along the loop2 region enhanced for high constitutively active mutants and decreased for low constitutively active mutants. Overall, we showed that the current levels of GoF TM3 mutants are not only determined by amino acids in TM3, but also by the entire loop2 region. We identified that constitutive currents increased with reduced hydrophobicity along TM3 and enhanced hydrophobicity along the loop2 region. We recently published that the loop2 region of Orai channels exhibits isoform-specific functional and structural features in respect to a co-regulation with the Orai N-terminus. MD simulations revealed that the loop2 region in Orai1 contains a longer helical portion than that of Orai3 (Fahrner et al., 2018). Thus, it seems that the lower flexibility of the loop2 region possesses a stimulating effect on Ca^2+^ permeation. Moreover, also Orai1-Orai3 chimeras clearly revealed that the synergy of the two non-conserved residues in TM3 maintains the closed state of the respective Orai variant. Reducing the overall hydrophobicity along TM3 by a swap of amino acids by the respective other Orai isoform leads to constitutive activity not only in Orai wild-type, but also in the Orai1-Orai3-L2 chimeras.

It is worth noting that these non-conserved hydrophobic residues in TM3 point to hydrophobic residues in TM4, suggesting that communication of TM3 and TM4 controls Orai activation (Tiffner et al., 2021). We recently reported that Orai1 F250C, located opposite L185, shows small constitutive activity (Tiffner et al., 2020a). Interestingly, while also Orai1 L185A/S show weak constitutive activity, we discovered that a double point mutant Orai1 L185A F250A exhibits strongly pronounced constitutive currents (Derler et al., 2018; Tiffner et al., 2020a). Moreover, it is known that P245L (Nesin et al., 2014; Palty et al., 2015; Derler et al., 2018), associated with the Stormorken syndrome and located at a kink in the middle of TM4, and the _261_ANSGA_265_ mutations (Zhou et al., 2016), located at the bent connection between TM4 and C-terminus, induce GoF. Despite this knowledge on TM3 and TM4 mutations, it remains to be determined how they affect the communication of TM3 and TM4 to maintain the closed state or to induce pore opening. Currently available dOrai structures suggest that pore opening involves a pore dilation and outward rigid body movement of all TM domains especially at the cytosolic side of the channel complex (Hou et al., 2012, 2018, 2020; Liu et al., 2019). Interestingly, the crystal structures of dOrai open states further resolved a straightening of the TM4-C-terminus region, which, however, is not visible in dOrai cryo-EM structures. Thus, further studies are required to resolve which conformational changes occur physiologically at the outmost side of the channel complex to induce Orai pore opening and whether they occur in an isoform-specific manner.

Summarizing, we discovered that non-conserved gating checkpoints in TM3 of Orai1 and Orai3 control the maintenance of the closed state and an opening permissive channel conformation in an isoform-specific manner, while overall global conformational TM motions are indispensable for pore opening of both channels. The extent of current size increases with reduced overall hydrophobicity along TM3 and enhanced hydrophobicity along the loop2 region. The elucidation of isoform-specific differences provides novel targets for the development of future therapeutic interventions in an Orai-isoform-specific manner.

## Data Availability Statement

The original contributions presented in the study are included in the article/Supplementary Material, further inquiries can be directed to the corresponding author/s.

## Author Contributions

AT, LM, and ID conceived and coordinated the study and wrote the manuscript. ID, AT, and LM performed and analyzed the electrophysiological experiments. ID performed the Secondary Structure Assignments and Bioinformatics. MF and CH contributed to molecular biology. HG performed the confocal localization measurements for Orai mutants. MS and HG performed the NFAT studies. MS analyzed the NFAT experiments. SW performed and analyzed the Ca^2+^ fluorescence measurements. All the authors reviewed the results and approved the final version of the manuscript.

## Conflict of Interest

The authors declare that the research was conducted in the absence of any commercial or financial relationships that could be construed as a potential conflict of interest.

## Footnotes

1 https://web.expasy.org/protscale/
