# Supplementary material for: Transmembrane Domain 3 (TM3) Governs Orai1 and Orai3 Pore Opening in an Isoform-Specific Manner
Source: Front Cell Dev Biol. 2021 Feb 11;9:635705. doi: 10.3389/fcell.2021.635705 (PMC7905104; doi:10.3389/fcell.2021.635705)
Supplement: Supplementary file 1 [file Data_Sheet_1.pdf]

Supplementary Material

Supplementary Figure 1:

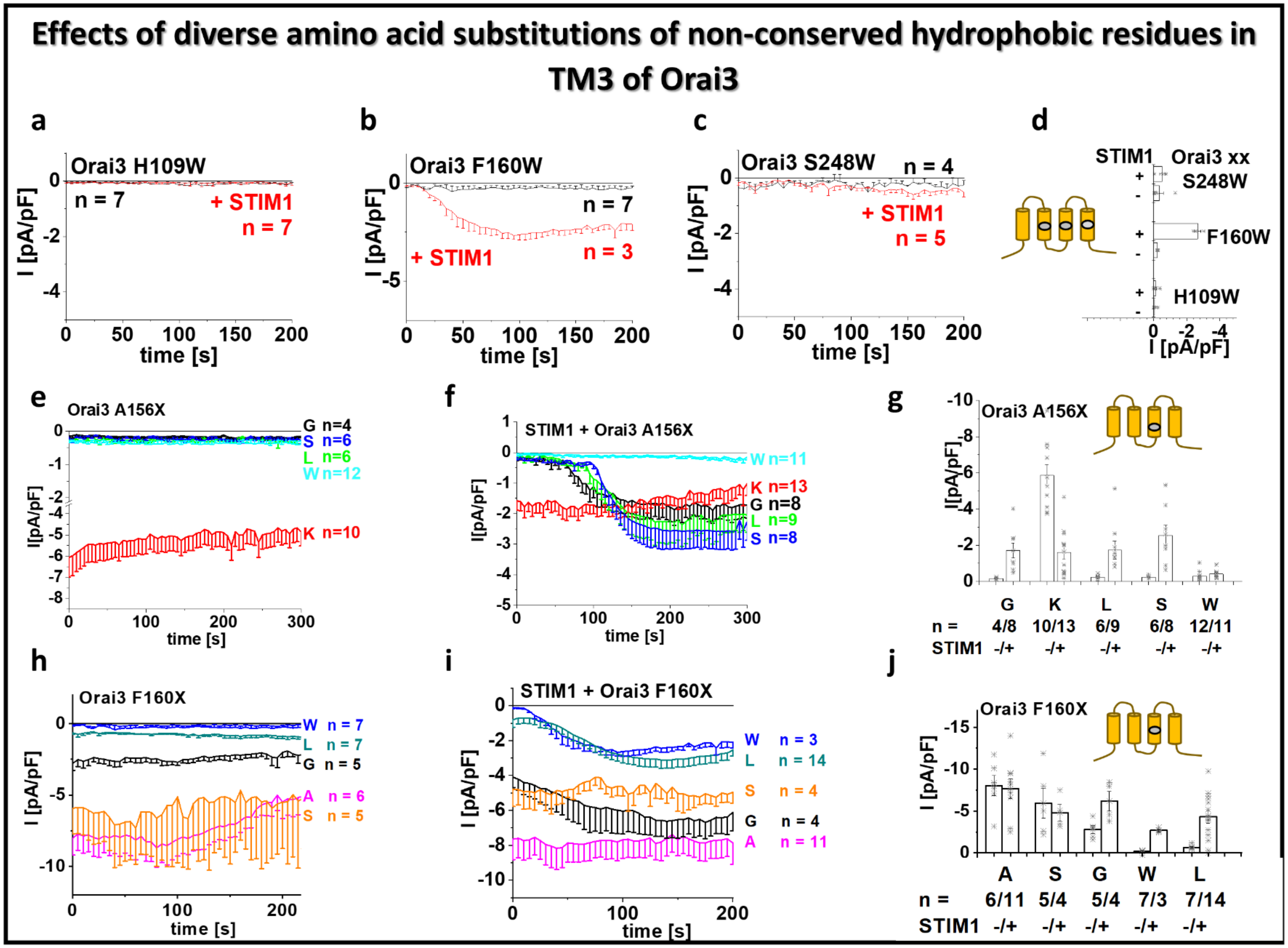

Supplementary Figure 1: Effects of diverse amino acid substitutions of non-conserved hydrophobic residues in TM3 of Orai3.
a-c) Time course of current densities after whole-cell break-in of loss-of-function Orai3 single point mutants Orai3 H109W (a), Orai3 F160W (b) or Orai3 S248W (c) in the absence and presence of STIM1. d) Block diagram with maximum current densities corresponding to data recorded in (a – c). e-f) Time courses of current densities after whole-cell break-in of Orai3 A156X (X = G, K, L, S, W) single-point mutants in the absence (e) and presence (f) of STIM1. g) Block diagram of whole-cell current densities recorded in (e-f). h-i) Time courses of current densities after whole-cell break-in of Orai3 F160X (X = A, G, L, S, W) single-point mutants in the absence (h) and presence (i) of STIM1. j) Block diagram of whole-cell current densities recorded in (h-i).
In (d), the Mann Whitney test was employed for statistical analyses with differences considered statistically significant at $p < 0.05$. Pairwise comparison of Orai3 mutant currents in the absence and presence of STIM1 revealed a significant difference for Orai3 F160W currents in the absence compared to the presence of STM1. In (g, j), the Welch-ANOVA test (due to lack of variance homogeneity as determined by Levene Test) was used for statistical comparison of Orai3 mutants using the F-distribution ($F(9,28.79) = 13.97$, $p < 0.001$ (g), $F(9,28.89) = 13.96$, $p <$ 0,001 (j)). Subsequent to Welch-ANOVA we performed the Games-Howell post-hoc test to determine the pairs which differ statistically significant ($p < 0.05$). Statistical significance was determined for Orai3 A156K compared to STIM1 + Orai3 A156K as well as to all other Orai3 A156X (X = G, L, S, W) point mutants, both in the absence and presence of STIM1 (g) and for Orai3 F160W compared to Orai3 F160A/S/G/L in the absence of STIM1 (j).

Supplementary Figure 2:

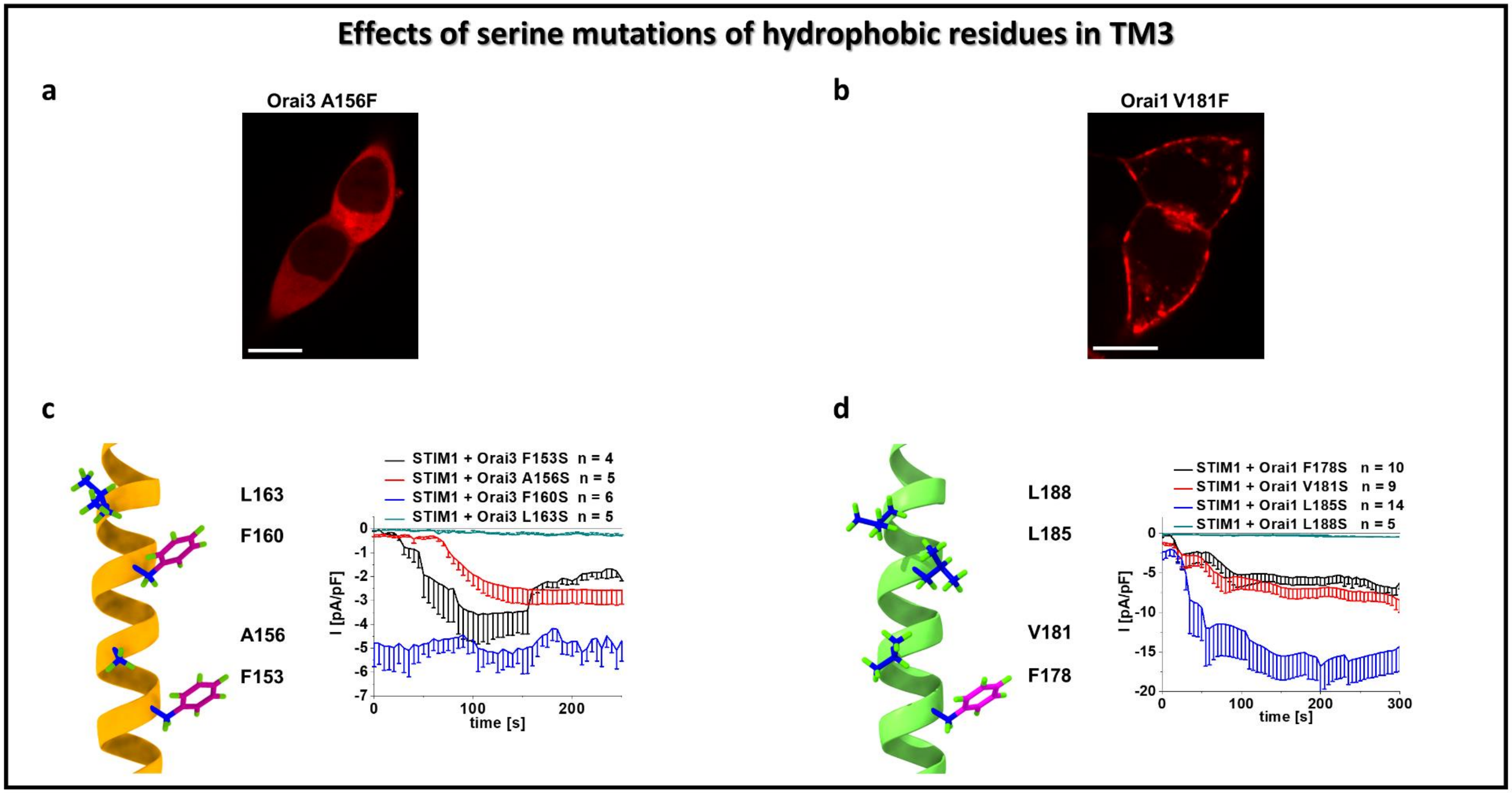

Supplementary Figure 2: Effects of serine mutations of hydrophobic residues in TM3 of Orai1 and Orai3.
a-b) Image series depict YFP-Orai3 A156F (a) and YFP-Orai1 V181F (b) mutants. Orai3 A156F displayed loss of PM localization, while the analogous mutant Orai1 V181F revealed intact PM trafficking. c) d) Schemes highlight the analogous residues of interest within this figure for Orai3 (orange, left) and Orai1 (green, middle). c) Time courses of current densities after whole-cell break-in of Orai3 F153S, Orai3 A156S, Orai3 F160S and Orai3 L163S in the presence of STIM1. d) Time courses of current densities after whole-cell break-in of Orai1 F178S, Orai1 V181S, Orai1 L185S and Orai1 L188S in the presence of STIM1.

Supplementary Figure 3:

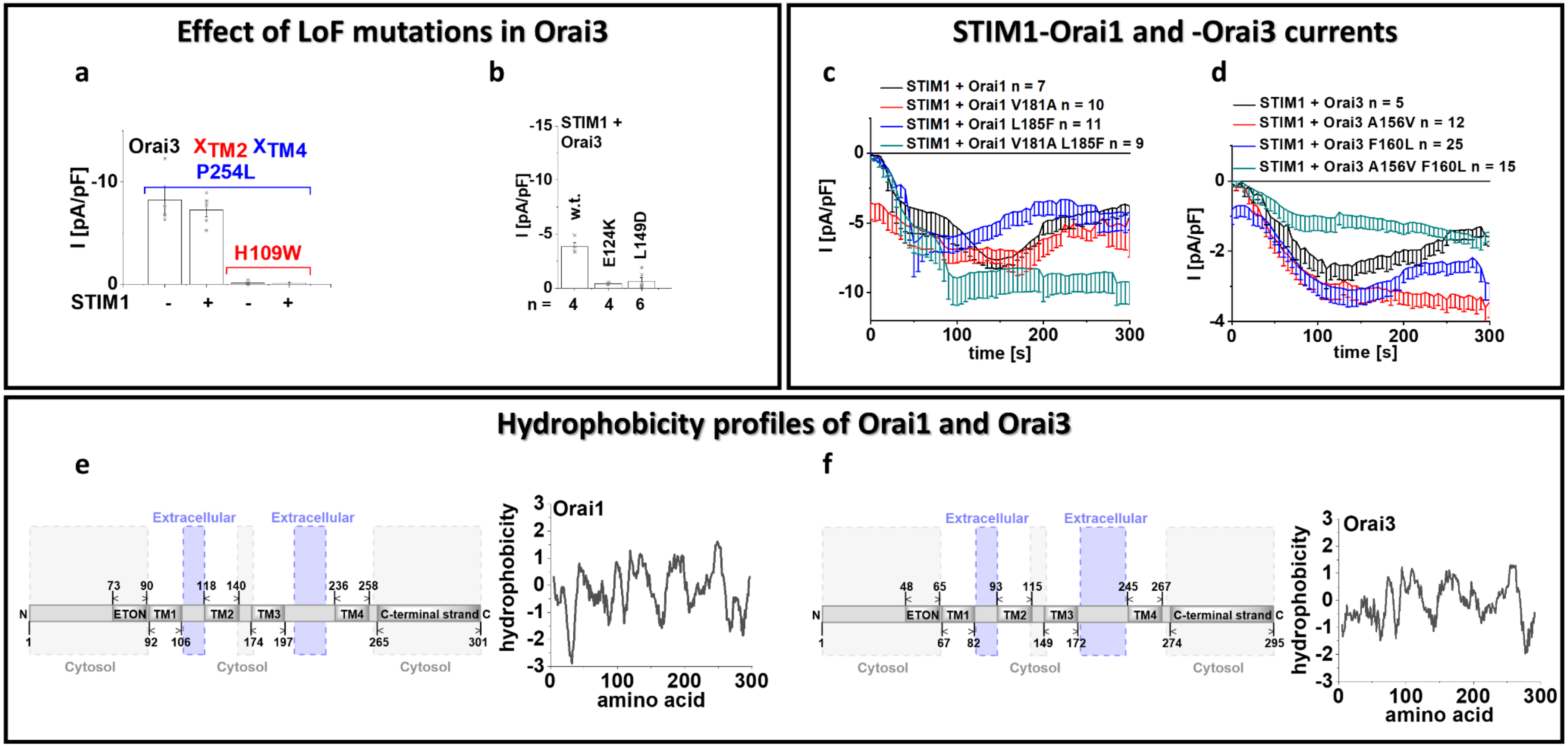

Supplementary Figure 3: Effects of diverse single and double Orai1 and Orai3 mutants and hydrophobicity plots of Orai1 and Orai3. a) Block diagram of whole-cell current densities after whole-cell break-in of Orai3 P254L and Orai3 H109W P254L in the absence (t = 0 s) and the presence (maximum current densities) of STIM1 (n = 4-5 cells; values are mean ± SEM). b) Block diagram of whole-cell current densities after whole-cell break-in of prominent LoF CETR mutants Orai3 E124K and Orai3 L149D compared to Orai3 in the presence of STIM1 (n = 4-6 cells; values are mean ± SEM). c) Time courses of current densities after whole-cell break-in of Orai1 V181A, Orai1 L185F and Orai1 V181A L185F (thus mimicking the positions A156 and F160 in Orai3) compared to Orai1, all in the presence of STIM1. d) Time courses of current densities after whole-cell break-in of Orai3 A156V, Orai3 F160L and Orai3 A156V F160L (thus mimicking the positions V181 and L185 in Orai1) compared to Orai3, all in the presence of STIM1. e-f) Scheme depicting the full-length Orai1 and Orai3 channel

with highlighted TM and loop regions pointing out the cytosolic and extracellular portions with their corresponding hydrophobicity profiles of Orai1 (e) and Orai3 (f) based on the Rose and Lesser hydrophobicity scale using the Expasy-Protscale prediction program (https://web.expasy.org/protscale/).

In (a, b) the Welch-ANOVA test (due to lack of variance homogeneity as determined by Levene Test) was used for statistical comparison of Orai3 mutants using the F-distribution ($F(3,6.97) = 41.66$, $p < 0.001$ (g), $F(2,5.16) = 38.95$, $p < 0,001$ (b)). Subsequent to Welch-ANOVA we performed the Games-Howell post-hoc test to determine the pairs which differ statistically significant ($p < 0.05$). Statistical significance was determined for Orai3 GoF mutant currents compared to Orai3 GoF-LoF double mutant currents (a) and STIM1-mediated Orai3 currents compared to those of Orai3 LoF mutants (b).

Supplementary Figure 4:

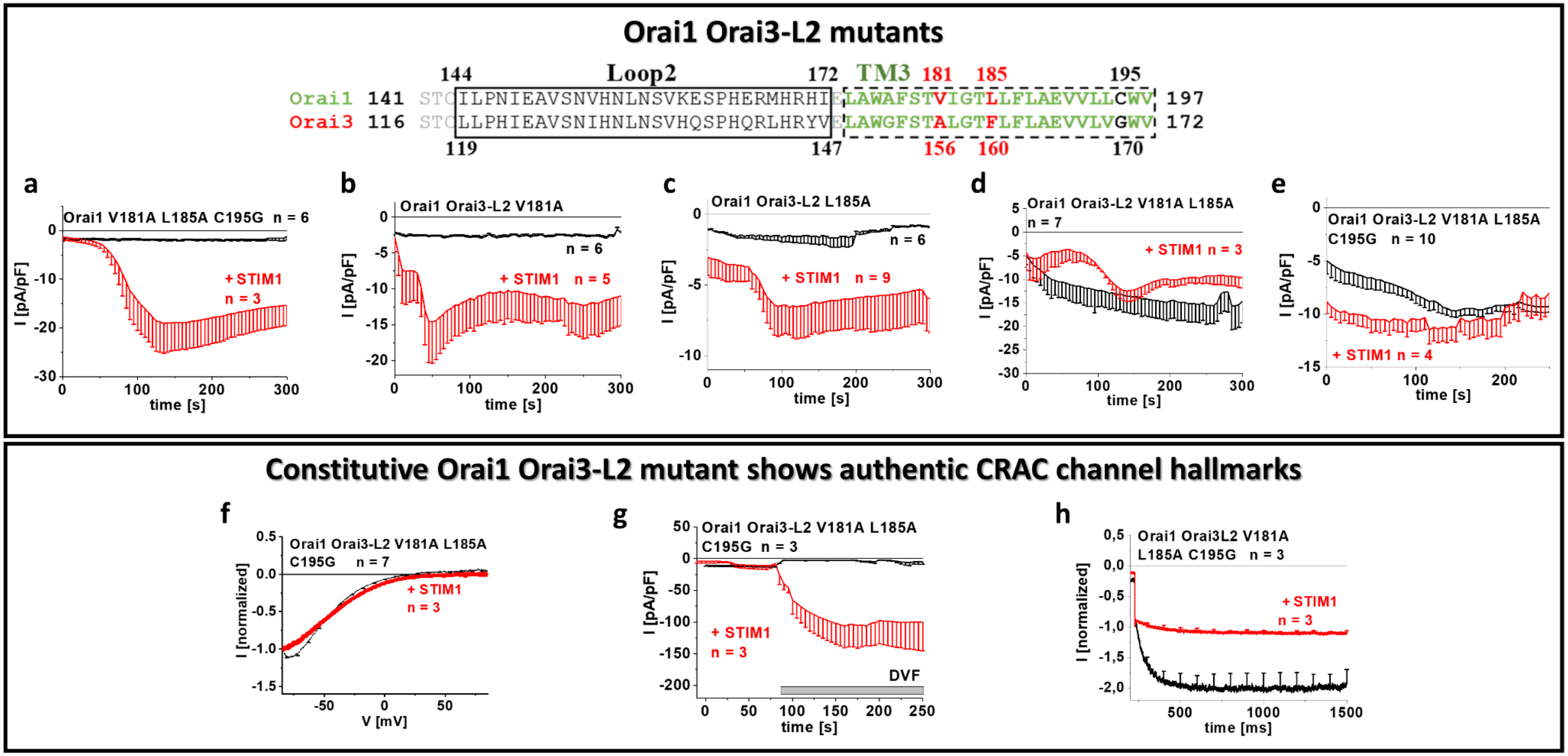

Supplementary Figure 4: Effects of diverse Orai1-Orai3-loop2 mutants.

The scheme represents the sequence alignment of Orai1 and Orai3 at the loop2 and TM3 region (middle). a-e) Time courses of current densities after whole-cell break-in of Orai1 V181A L185A C195G (a), Orai1 Orai3-L2 V181A (b), Orai1 Orai3-L2 L185A (c), Orai1 Orai3-L2 V181A L185A (d) and Orai1 Orai3-L2 V181A L185A C195G (e) in the absence compared to the presence of STIM1. f) Normalized I/V relationships of Orai1 Orai3-L2 V181A L185A C195G in the absence compared to the presence of STIM1. g) Time courses of current densities after whole-cell break-in of Orai1 Orai3-L2 V181A L185A C195G in the absence and presence of STIM1 upon application of a Na^+-containing DVF solution. h) Inactivation characteristics of Orai1 Orai3-L2 V181A L185A C195G in the absence compared to the presence of STIM1.
